# Doctors, Quack Doctors, and Doctor Quacks

**Published:** 1837

**Authors:** 


### II.—ANALYTICAL.

I.—Doctors, Quack-Doctors, and Doctor-Quacks. *The Newspapers, of the United States, for* 1837.

The whole medical profession, that is, all who profess medicine, for in no other sense can the term be applied in the United States, may be divided into three classes—Doctors, Quack-Doctors and Doctor-Quacks. The first comprises men of science, who respect themselves and the dignity of the calling, which they follow; the second have neither science nor self respect, and rove through the nation with commissions of piracy; the third have a *portion* of both—being doctors and quacks at the same time, though the latter is sometimes in so small a quantity, as to impart, but few *qualities* to the *compound.* The generic characters, of the first division, may be expressed by the words—*learned, studious, humane, honorable;* and to this class, we hope all, who are of a pugnacious temper will betake themselves; that we may not be involved in a "doctor's-quarrel," a matter for which we are well known to have a most settled aversion. That freedom of the press, under which we indite this piece of natural history, is the glory of our union. But we, Americans, are not the first people who have gloried in their shame. If all the vituperation, slander, falsehood, and obscenity to which the American press gives utterance, were pressed out of what is published, there would be little left, except a *caput mortuum.* But of course no body will believe this, for every body holds, that we Americans, are the most moral and enlightened people on the earth—that one half are too good to practice an imposture, and the other too wise to be duped—and, (two halves making a whole,) that we are, in the aggregate, an admirable nation. For ourselves, however, while out of deference to the people, we retract all that is here put forth, we must be permitted to say, that we believe more than half of it to be true.

It is in the great cities of the EAST, more than elsewhere, that the press has furnished a magnificent common sewer, for the *effusions* of the tens of thousands, who pay for admission into it, out of the hard earnings of the credulous people on whom they pour out their *balms*, *extracts*, *balsams*, *and liniments;* of such *aperient* virtues, as to open every *sphincter bursæ;* which, liberally translated, signifies to pick the pocket of every fool. But the WEST is not behindhand in these gratifying results of the art of printing; and Cincinnati, Louisville and St. Louis, although young and feeble in civic organization, are old and powerful in their contributions to the sluices of medical imposition.

Of our second division the quack doctors, who empty their bottles of *infallible panacea* into this polluted stream, we shall not say a great deal. They are too popular with all classes of people to be assailed with impunity; and, as we sincerely desire to stand well with the intelligent and orderly part of society, we shall not call in question their taste and judgment; nor speak of their gullibility when they gulp down *vegetable specifics*, or the *essential oil of Madagascar bats: de gustibus non disputandum.* It is a bad calculation to run in opposition to ones betters; and he who spits against the wind soils his own face. It is only when the steam boat braves the current, that she gets snagged; and, in a republican government, he who keeps with the majority is of course right. We say, therefore, to those gentlemen and ladies, scholars, editors, lawyers, divines, professors and missionaries, who are in the daily ingurgitation of the pills and bolusses, and linctuses, and essences of roots, and conserves of *botanical plants*, prepared for their especial consumption, by Indian doctors and others—*be ye filled!* It is tiresome to be always practicing the lady or gentleman; and, equally irksome, to be always governed by the maxims of common sense. Folly is the pass-time of wisdom; vulgarity the relaxation of dignity, and both should be enjoyed. Equality, moreover, is the vital principle of a republic, and there *should* be high-ways, on which the learned and ignorant, the rude and genteel, the rich and poor, can move on arm in arm; and what road more proper than that which leads to the shop of the quack-doctor? The grave levels all distinctions, and why should not its laboratory do the same? Again we say, down with monopolies and let all classes have an equal chance! It is unjust to require a gentleman to pay a physician's bill, when he can cure himself with twenty-five cents worth of patent pills—it is ungallant to compel a delicate lady to employ a man of sense, when her taste is for vagabonds.

Our chief object, however, is not to defend the ladies nor the quack-doctors; for their mutual support renders a defence unnecessary. Nevertheless, we cannot refrain from adding, that we admire both—one set for their credulous and amiable confidence: the other, for that reckless impudence, which raises them into the rank of heroes and'corsairs, and thus secures the admiration and patronage of the fair. Having borne this testimony to the merits of both parties, we shall leave them to themselves and to each other, and proceed to speak of our third division—the *doctor-quacks*.

These, as the name imports, are *learned* men. When Pope declared—

> "*A little learning is a dangerous thing*"—

he doubtless applied it to the individuals themselves; in the practice of physic, however its applicability is to the patient, rather than the physician. But to designate the doctor-quack as dangerous to the community, is not to distinguish him sufficiently from other classes. He is a wolf in sheep's clothing—for it is his "sheep-skin"—the only object of *ambition* he ever had—that first gave him currency, and afterwards kept him in the fold. The want of this, keeps out the quack-doctor; and herein lies another distinctive mark of the two varieties. Would you strike a blow upon him, he is protected by his wool. It is melancholy that the skin of a sporting and innocent lamb should be devoted to such a vile purpose. The bed of Procrustus was, beyond a doubt, made of sheep-skins, for they can stretch the ignoramus into a philosopher. Contemplate the doctor-quack with his roll of embellished parchment in one hand, and he stands before you in all the dignity of learning—look at the glaring catalogue of nostrums, which he displays from the other, and you register him with the herd of quacks; thus he is the connecting link between two extremes, a professional hybrid; but his mulattoism does not, as in other cases, sink him from among the sons of light. Would you cast him down from the high and hallowed eminence on which men of science and honor congregate, his "sheep-skin" buoys him up;—would you make war upon him, he unfolds it as a banner, and the people flock to his support. Would you even devote to his professional history a page of the WESTERN JOURNAL—you are jealous of his popularity, a libeller, a persecutor! and not coveting these epithets we shall speak of him with caution.

The doctor-quack is of *all* countries, and, therefore, of our own beloved *West;* the land, *par excellence*, of enterprize, versatility, freedom of action, boasting, and admiration of bold imposture. The land, where nominal diversities are brought into juxta-position; where the doctor-quack, the quack-doctor and the farrier, address the people in the same vocabulary, and grease and funnel them, with the same compounds of words, if not of drugs. Happy people, to have within their reach, at a dollar a bottle, all that man or beast can desire in the hours of sickness and sorrow. Let us cut from the newspapers and send abroad through a new channel some of their comforting annunciations.

### Several Doctor Quack's Advertisements.

### GALEN'S PILLS AND PLASTERS:

NEW REMEDIES FOR

### CHRONIC DISEASES, INCLUDING ALL THE FORMS OF SCROFULA.

THE attention of the public is respectfully invited to the new remedies for Scrofulous diseases. These remedies, with a few exception in the last stage, have never failed to cure all the cases of the disease properly called, *Chronic Turbercula of the Organs and Limbs.* To wit:

| | | | | |
|---|---|---|---|---|
| Consumption, | Chronic | Turbercula | of the | Lungs. |
| Dpspepsia, | " | " | " | Stomach. |
| Liver Complaint | " | " | " | Liver. |
| King's Evil | " | " | " | Neck. |
| Mercurial disease | " | " | " | Tonsils, palate and tongue. |
| Scrofulous sore eyes, | | " | " | Eyes. |
| Scald head | " | " | " | Scalp. |
| White swellings | " | " | " | Joints and Limbs. |
| Leucrrohoæ } | | " | " | |
| Chlorosis and } | | " | " | Uterus. |
| Menorrhagia, } | | " | " | |
| Chronic Diarrhoæ | | " | " | Mesentery. |

Diseases of the spine, etc.

That these are all cases of the same disease, affecting the different organs and limbs, exhibiting the same symptoms and dissections, and consequently cured by the same remedies, is fully demonstrated in the pamphlet which accompany each box of the remedies, entitled, 'New and Natural Symptoms, and New and Natural Remedies in Chronic Diseases,' founded on the natural causes of motion in animate matter, by which the practice is greatly simplified, and a knowledge of it easily attained. These remedies require no dieting while taking them, and never produce any disagreeable effect upon the stomach or bowels; nor does the patient perceive any effect while using them, except a steady improvement in health and strength, and in all the symptoms dependant on the disease.

*** There is no mistake about the new symptoms, causes of motion or remedies: they have been fully tested. The new symptoms are positive indications of the disease, and the remedies are as certain to cure the same as Sulphate of Quinine is to cure ague.

FAMILY MEDICINES.—The proprietor having been extensively engaged in the practice of medicine for nearly twenty years, witnessing the impositions practiced [upon the public in the sale and use of various Nostrums and Patent Medicines, by which there has been a great sacrifice of property, health and life; and feeling the want of a proper assortment of Family Medicines, is induced to offer the following preparations, with the assurance that they are not only safe, but when taken in season, and with due care and attention, will effectually free the system of disease, without injury to the constitution.

The proprietor has long been in the use of the medicines now offered, and feels confident that they are worthy of publlc patronage.—They are such as he has been in the habit of prescribing in his private practices, and such as he does not hesitate to recommend to his medical brethren. He might have obtained volumes of certificates of the utility and efficacy of his medicines; but the public have been so often deceived and cheated both out of their money and health by means of certificates, that they have ceased to be worthy of public attention

THE MEDICINES consist of two kinds of Pills. No. 1, Cathartic, No. 2, Laxative. An Anodyne Liniment, for Rheumatisms, Sprains, Local Pains, etc. A cordial for Coughs, Colds, Lung, Nervous complaints, and for Children. A Worm Powder. An Ointment for diseases of the skin.

☞ Physicians are particularly invited to give the above medicines a trial.

TONIC AND ANTI-DYSPEPTIC PILLS.—This Medicine is offered to the public with a confidence produced by long experience in practice, that they possess the following properties. They will operate greatly as a purgative, and as effectually cleanse the stomach and Intestines of all offensive and irritating matter as any medicine now known, and will not debilitate them. They do not produce the least sickness or nausea, but on the contrary, the appetite and feelings will generally be improved in one hour after the Pills are taken. They can be used at all times, and by all ages. No attention is necessary to diet, drink, or exposure to wet or cold, while using them. A few boxes will be found generally to remove the most confirmed Dyspepsia, with all its distressing symptoms.

### DOCTOR BLANK'S MEDICINES.

THE subscriber has become Agent for the sale of the very valuable Medicine, prepared by Doctor Blank, and will hereafter keep a constant supply of them, viz:

Alterative Pills, to correct all derangements of the stomach, liver, and other digestive organs.

Anti-cholera Pills, a speedy and effectual cure for cholera in its first stages, cholera morbus, and diarrhœa, or bowel complaint.

Ague Pills, a warranted cure for the ague and fever, and other intermittent diseases.

Anti-dysenteric Pill, for the dysentery or bloody flux.

Anti-acid Pills, for sour stomach, heart-burn, and sick headache.

Active Cathartic Pills, a quick and thorough purgative.

Powders for weakly Females.

Medicated Balsam,

Restorative Mixture,

These are safe and effective remedies for the diseases peculiar to females.

These important medicines have not been puffed into use, as it is customary to do, with empirical preparations, by publishing certificates and recommendations, although abundance of those of the first character could be obtained; but they have been left silently to gain a hold on the confidence of the public, by a long experience of their superior efficacy. Their best recommendation is that few or none who have once used them, ever lose their confidence in them, or seek other remedies for those diseases for which these are prescribed.

### GENUINE SPECIFIC OINTMENT.

THE ONLY TRUE AND GENUINE

### DR. BLANK'S SPECIFIC OINTMENT.

FOR the cure of White Swellings, Sore Legs and Ulcers, Glandular Tumors, Felons, Rheumatic Pains, Sprains and Bruises, Burns and Scalds, Inflammation in Women's Breasts Glandular Swellings, Old and Fresh Wounds, Chilblains, Tetters, Piles, Ring Worms, Eruptions of long standing, and sore eyes.

I have produced a few certificates of cures, to promote its utility; and some of them from members of Congress of the United States, as well as the very first scientific characters in the country, besides a number of my medical brethren; and when I turn and examine the evidence which has been given in such flattering terms, as to the many extraordinary cures of diseases of long and painful standing, and which had resisted many ordinary remedies, administered by skillful hands. I feel grateful in finding myself the instrument, and thus handing out such remedial agent by the effects of which, so much human suffering has been erased; and all I wish or desire, is that those who may be afflicted with any of the above diseases may give it a fair trial for themselves, and then they will be fully competent to judge correctly of its utility.

—

Three or four Quack-Doctor's Advertisements.

—

### THE VEGETABLE PULMONARY BALSAM.

THE most valuable remedy discovered for Consumptions, Coughs, Colds, Asthma, Spitting of Blood, Hooping Cough, and Pulmonary Affections of ever kind.

The great celebrity of the *Genuine Vegetable Pulmonary Balsam* has been the cause of attempts to introduce spurious articles, which by partially assuming the name of the genuine, are calculated to mislead and deceive the public. Among these mixtures are the "American Pulmonary Balsam," "Vegetable Pulmonary Balsamic Syrup," and others. Purchasers should inquire for the TRUE article by its WHOLE name, *The Vegetable Pulmonary Balsam,* and see that it has the marks and signature of the genuine.

Each bottle and seal is stamped VEGETABLE PULMONARY BALSAM.—Signed SAMPSON REED.

### ANDREW'S HIGHLY VALUABLE CIRCASSIAN EXTRACT,

FOR promoting the growth and preservation of the Hair. Prepared in vacuo, from Milleferous and Balsamic Products of the East.

This elegant and valuable article is prepared upon truly philosophical and chemical principles, to counteract the effect of the hair turning grey, by promoting its growth, and producing a vigorous action in the capillary tubes; obtaining all that can be desired for the preservation of the hair.

Where the powers of nature for the re-production of the hair have not totally receded, this desirable preparation has also been found, to a most extraordinary degree, successful in arresting the progress of baldness, even in persons of advanced age.

The most arduous researches have been displayed, and a series of most splendid efforts have been made by chemical experiment, to unfold the mysteries of nature, so as to place this extract beyond all doubt and comparison, superior to every preparation hitherto brought before the public.

### DR. BURNHAM'S DROPS, OR MEDICINE FOR FEVER.

AS anti-bilious and anti-dyspeptic Medicine, the DROPS are unrivalled, where they are known, by any thing yet recommended.—Persons who have suffered for years with pain in the side—breast—stomach or lungs—and when, to all appearances, disease had made extensive ravages, and seemed to be beyond the control of medicine, have been in all cases of trial greatly benefitted; and in a large majority of instances entirely relieved—as the cases of Mrs. Hunter, of Lancaster, Miss Burwell, of Columbus, and Mrs. Tiffany, of Madison, abundantly testify—and many others not here enumerated, who have been relieved from the sufferings of periodical sick, or nervous headache, billious and cramp cholic.

### GRAY'S INVALUABLE OINTMENT.

FOR THE CURE OF

WHITE Swellings, Scrofulous and other Tumours, Ulcers, Sore Legs, old and fresh Wounds, Sprains and Bruises, Swellings and Inflammations, Scald Head, Women's Sore Breasts, Rheumatic Pains, Eruptions, Chilblains, Whitlows, Bites, Piles, Corns, and external diseases generally.

—

### A Horse-Doctor's Advertisement.

—

### GARDNER'S CELEBRATED VEGETABLE LINIMENT.

FOR THE CURE OF

SPRAINS, Bruises, Cuts or Wounds; Corks, Chaiffs or Galls; Film in the eye, and every external complaint to which Horses are liable. Also burns or scalds, fresh wounds, Rheumatic pains, Swelling of the Glands of the throat or Croup, Ague in the Face, Ring worms and Tetter, painful Tumors, etc.

### NOTICE.

It is now about five years since I first discovered this valuable remedy—during which time I have prepared it chiefly for my own use, and that of my immediate acquaintances in curing the external complaints of Horses. Knowing full well that the people are daily imposed upon by the immense flood of *nostrums* forced them, that they have been deceived and have become disgusted with every thing like *quackery*, I for a long time hesitated to bring the above named article forward, but am thus forced upon to do it by the repeated, urging and constant advice of those to whom I have distributed the article for the cure of several complaints mentioned above. They have *unsolicited*, presented me with their certificates for publication, that others may be benefitted, some of which I present.

JAMES GARDNER.

Now we call upon the profession to say, whether all the specimens here given, are not in the same spirit and letter; and whether the physician, the quack and the farrier, do not combine themselves into one natural group? If there is any difference in the character of their advertisements, those from members of the profession, the doctor-quacks, are most in violation of propriety—most incompatible with sound science--most delusive and most pernicious, because they go forth under the sanction of titled names. These names we have suppressed, as we deal with acts and not persons. Towards the individuals themselves, as far as personally known to us, we cherish no unkind feelings, and do not doubt, that in many respects, they are qualified to practice medicine on scientific principles. This, however, but increases our regret, and the disposition to arrest them in their wanderings from the path of duty. In theory, they themselves must condemn such aberrations into the region of empyricism; but, alas for poor human nature, how easily may it be bribed into the practice of what it abhors! alas for the honor of a profession, which can exercise over its members so little restraint! alas for a community, whose longings are best satisfied with vegetable incantations and cabalistic unguents! alas for him who would stand between quackery and its victims!

### II.—Patronage of Quacks.

The *London Medico-Chirurgical Review* for Oct. 1836, has a page under this title. The learned editor, Dr. Johnson, seems but recently to have met with the catagory of distinguished names, which testify to the transcendent virtues of Swaim's Panacea. He has immortalized them in his widely circulated journal; for this we cannot blame him, but as *Americans*, we are kept from recording them in our humble quarterly. They are, indeed, already but too well known throughout the land. When the medical dignitaries of the enlightened *East*, can allow themselves to stoop so low, what may not be tolerated in the Backwoods! The stream cannot rise above its fountain—but let us come to the extract.

### Patronage of Quacks.

We thought that in England, quackery flourished with unexampled vigor. But we were mistaken. It is rarely that the quack here is able to entrap the regular physician or surgeon into an open patron, certifying under his hand and seal the mighty virtues of an unknown compositition, vended for gain, and for gain alone. Not so beyond the Atlantic! We were utterly astonished to find an impudent panacea bolstered up with the names and certificates of some of the first authorities, in the medical profession of the United State! We were thunderstruck on perusing such documents as the following—some twenty or thirty being obtained by a noted nostrum-vender in the land of freedom.

Here come in the certificates of four distinguished medical gentlemen of Philadelphia and New York, which a sense of shame will not permit us to copy.

Eheu, jam satis! We are mortified and grieved beyond measure, to find professional *propriety* (to give it no other name) at so low an ebb among our brethren in America! This admonition from Europe will surely rouse the faculty of the United States to some sense of the duty they owe to their brethren throughout the world.

